# Circulating Biomarkers in Elderly Patients with Heart Failure: A Real-Life Study

**DOI:** 10.3390/antiox15030305

**Published:** 2026-02-28

**Authors:** Velia Cassano, Caterina Gabriele, Maria Rosangela Scarcelli, Giuseppe Armentaro, Giandomenico Severini, Domenico Martire, Carlo Alberto Pastura, Sofia Miceli, Marta Letizia Hribal, Giuseppe Massimo Claudio Rosano, Marco Gaspari, Angela Sciacqua

**Affiliations:** 1 Department of Medical and Surgical Sciences, University Magna Graecia of Catanzaro, 88100 Catanzaro, Italy; mariarosangela.scarcelli@studenti.unicz.it (M.R.S.); giandomenicoseverini@gmail.com (G.S.); domenico.martire98@gmail.com (D.M.); carloalbertopastura@gmail.com (C.A.P.); hribal@unicz.it (M.L.H.); sciacqua@unicz.it (A.S.); 2 Department of Experimental and Clinical Medicine, University Magna Graecia of Catanzaro, 88100 Catanzaro, Italy; cgabriele86@gmail.com (C.G.); gaspari@unicz.it (M.G.); 3 Geriatrics Division, “Renato Dulbecco” University Hospital of Catanzaro, 88100 Catanzaro, Italy; giuseppearmentaro91@gmail.com (G.A.); sofy.miceli@libero.it (S.M.); 4 Department of Human Sciences and Promotion of Quality of Life, San Raffaele Open, University of Rome, 00185 Rome, Italy; giuseppe.rosano@gmail.com

**Keywords:** biomarkers, elderly, heart failure, proteomic

## Abstract

**Background**: Heart failure (HF) is a clinical syndrome that involves multiple interconnected pathways. Circulating biomarkers in HF emerged as powerful tools for risk stratification, diagnostic confirmation, prognostic assessment, and monitoring of treatment efficacy. The aim of the present study was to evaluate circulating levels of biomarkers in elderly patients with improved HF ejection fraction, previously with left ventricular ejection fraction (LVEF) <40%, after six months of drug therapy optimisation. **Methods**: We enrolled 100 HFimpEF outpatients. All patients provided medical history and underwent physical examination at baseline and after six months of follow-up. The serum values of circulating biomarkers were assessed with an ELISA test. Proteomic analysis was performed on serum samples collected from a subset of 13 patients at baseline and after six months of follow-up. **Results**: At follow-up, we observed significant improvements in glycometabolic, renal and inflammatory profiles (*p* < 0.001). Proteomic analysis revealed selective changes in key cardiovascular (CV)-related proteins, such as insulin-like growth factor-binding protein 4 (IBP4), thrombospondin-4 (TSP4), intercellular adhesion molecule 1 (ICAM1), and syndecan-4 (SDC4). **Conclusions**: This study demonstrates significant improvements across multiple CV biomarkers after six months of therapy optimisation in HFimpEF patients, providing evidence for comprehensive therapeutic effects targeting inflammation, oxidative stress, neurohormonal activation, and thrombotic risk.

## 1. Background

Heart failure (HF) is a complex clinical syndrome characterised by structural and/or functional cardiac anomaly and remains a leading cause of morbidity and mortality worldwide, affecting approximately 64 million people globally and imposing a substantial burden on healthcare systems [1,2]. HF can be classified, according to LVEF, as heart failure with reduced ejection fraction (HFrEF), heart failure with preserved ejection fraction (HFpEF), or heart failure with mildly reduced ejection fraction (HFmrEF) [3]. While the pathophysiology of HFpEF is multifactorial, HFrEF is characterised by an imbalance in the neuroendocrine systems regulating cardiovascular (CV) homeostasis [4]. In addition, a new clinical phenotype, HF with improved EF (HFimpEF), characterised by a previously reduced left ventricular ejection fraction (<40%) that subsequently improves to over 40%, has been observed in recent years [5].

The concept of HFimpEF identifies patients who exhibit an increase in LVEF over time and/or following pharmacological and device-based therapies. However, the distinction between improvement, remission, and recovery remains inconsistently defined and is primarily LVEF-centric, lacking the prognostic implications of these phenotypic transitions. Persistent knowledge gaps regarding long-term management and optimal therapeutic strategies underscore the need for further research to refine risk stratification and evidence-based decision-making in HFimpEF [6].

In this context, the identification of biomarker patterns in patients with HFimpEF could help to understand the biological remodelling associated with LVEF improvement.

The pathophysiology of HF involves multiple interconnected mechanisms, including neurohormonal activation, inflammatory responses, oxidative stress, myocardial remodelling, and metabolic dysfunction [3]. These complex pathways generate numerous circulating molecules that can serve as biomarkers, providing valuable insights into disease pathogenesis, progression, and therapeutic response. Circulating biomarkers in HF have emerged as powerful tools for risk stratification, diagnostic confirmation, prognostic assessment, and monitoring of treatment efficacy [4].

In HF clinical practice, the most established biomarkers include natriuretic peptides, in particular B-type natriuretic peptide (BNP) and N-terminal pro-B-type natriuretic peptide (NT-proBNP), which reflect cardiac wall stress and volume overload [7]. However, the clinical utility of these biomarkers can be limited by various factors including age, renal function, obesity, and comorbidities, necessitating the exploration of additional biomarkers that provide complementary information [8,9].

Recently, advances in high-throughput technologies and systems biology approaches have facilitated the identification of novel circulating biomarkers reflecting different pathophysiological processes in heart failure. These biomarkers can be categorised based on their biological origin and functional significance in HF pathogenesis.

In recent years, many studies have focused their attention on systemic mediators such as oxidative stress, myocardial injury, inflammation, fibrosis, and metabolic dysfunction [10].

Moreover, large-scale proteomic studies have identified several protein biomarkers associated with HF outcomes.

The integration of multiple biomarkers into multi-marker strategies has shown potential for improving diagnostic accuracy and prognostic performance compared to single biomarker approaches [11].

The aim of the present study was to evaluate the circulating levels of biomarkers in elderly patients with HFimpEF, after six months of drug therapy optimisation.

## 2. Methods

### 2.1. Study Population

In this observational, prospective, single-centre study, we enrolled 100 outpatients (87 males and 13 females, mean age 66.7 ± 11.8 years) referred to the Geriatric Unit of the University Hospital “Renato Dulbecco” of Catanzaro and the “Magna Graecia” University of Catanzaro. All patients presented with a diagnosis of HFimpEF according to ESC guidelines. Exclusion criteria included: chronic kidney disease stage IV K-DOQI (eGFR < 30 mL/min/1.73 m^2^, CKD-EPI), severe hepatic impairment (Child-Pugh Class C), history of angioedema, previous diagnosis of dementia or serious psychiatric disorders. Clinical evaluation, laboratory tests, ECG and colour Doppler echocardiogram were conducted at baseline and after 6 months of follow-up.

The protocol was approved by the University Ethics Committee (2022.384), and written informed consent was obtained from all participants in the “MAgna GraecIa evaluation of Comorbidities in patients with Heart Failure (MAGIC-HF)” study (ClinicalTrials.gov identifier: NCT05915364) and by the local Ethics Committee of Calabria Region, Italy (Catanzaro, Italy, document n. 304—20 October 2022). This study met the standards of good clinical practice (GCP) and the principles of the Declaration of Helsinki.

### 2.2. Study Procedures

All patients underwent therapeutic optimisation, if possible, with the introduction and/or increase in ARNI dosage, and the introduction of SGLT2i and MRA (Table 1).

At baseline and after 6 months of follow-up from therapeutic optimisation, all patients underwent an accurate medical history and complete physical examination with the determination of the main hemodynamic and anthropometric parameters. Comorbidities and drug therapies were also recorded. Evaluation of the NYHA functional class was carried out as suggested by current guidelines [3].

All patients underwent a 12-lead electrocardiogram (ECG), blood chemistry tests and a full echocardiogram-colour-Doppler.

The evaluation of clinical Blood Pressure (BP) was performed according to current guidelines. Measurements of BP were acquired in the left arm of patients in sitting position using a semi-automatic sphygmomanometer (OMRON, M7 Intelli IT, Omron, Milan, Italy) after five min of rest. BP values were the average of three measurements. This evaluation was repeated on three different occasions at least 2 weeks apart. Subjects with a clinic SBP > 140 mmHg and/or DBP > 90 mmHg were defined as hypertensive [12]. Pulse pressure (PP) values were acquired as the difference between systolic and diastolic BP measurements.

### 2.3. Echocardiographic Parameters

Echocardiographic recordings were performed using a VIVID E-95 ultrasound system (GE Technologies, Milwaukee, WI, USA) with a 2.5 MHz transducer. All patients were examined at rest and in the left lateral decubitus position. Measurements were obtained according to the recommendations of the American Society of Echocardiography [13], and the echocardiographic examinations were carried out by the same expert operator. Left ventricular mass (LVM) was calculated using the formula proposed by Devereux and corrected for body surface area (BSA), to derive the LVM index (LVMI) [14]. Among the parameters of left ventricular global systolic function, left ventricular ejection fraction (LVEF) and cardiac index (CI) were evaluated [13]. LVEF was calculated by the Simpson biplane method. Right ventricular systolic parameters were also measured by estimating the systolic pulmonary arterial pressure (S-PAP) [15].

The diameter of the right ventricular outflow tract (RVOT) and the right atrium area (RAA) were obtained according to ASE recommendations [13]. The movement of the tricuspid annulus was recorded at the free wall of the RV for the tricuspid annular plane systolic excursion (TAPSE), which expresses the right longitudinal function. In addition, for a more complete assessment of right ventricular function, the TAPSE/S-PAP ratio, an index of the right ventricular length/strength relationship, was calculated.

A 2D speckle tracking analysis was retrospectively performed using vendor-specific 2D speckle tracking software (EchoPAC PC, version 113.0.5, GE Healthcare, Horten, Norway). Manual tracings of the endocardial border during end-systole in three apical views was performed to evaluate global longitudinal strain (GLS) [16].

### 2.4. Laboratory Determinations

All laboratory measurements were performed after a fast of at least 12 h. Plasma glucose was measured by the glucose oxidation method (Beckman Glucose Analyzer II; Beckman Instruments, Milan, Italy), and plasma insulin concentration was determined by a chemiluminescence-based assay (Roche Diagnostics). Triglycerides, total, LDL, and HDL cholesterol concentrations were measured by enzymatic methods (Roche Diagnostics, Mannheim, Germany). Values of estimated glomerular filtration rate (e-GFR) were calculated by using the equation proposed by investigators in the chronic kidney disease epidemiology (CKD-EPI) collaboration [17].

### 2.5. Circulating Biomarkers Evaluation

Circulating biomarkers evaluation was performed on 100 enrolled patients at baseline and after 6 months of follow-up.

Blood samples, obtained from fasted patients, were taken in tubes with separator gel and centrifuged at 3000 rpm for 15 min to obtain serum samples that were immediately stored at −80 °C.

Quantitative determination of the oxidative stress serum biomarkers 8-isoprostane (ELISA kit Cayman Chemical, Ann Arbor, MI, USA) and Nox-2 (ELISA kit MyBioSource, San Diego, CA, USA) was performed with commercial ELISA immunoassays according to the manufacturer’s instructions. Values of 8-isoprostane were expressed as pg/mL; the lower detection limit of the assay was 0.8 pg/mL. Values of Nox-2 were expressed as nmol/L; the lower detection limit of the assay was 0.25 nmol/L. The coefficient of variation (CV %) was <9.0% intra-assay and <11.0% inter-assay [18].

Quantitative determination of platelet activation serum biomarkers sP-selectin and Glycoprotein-VI (GP-VI) (ELISA kit MyBioSource, San Diego, CA, USA) was performed with commercial ELISA immunoassays according to the manufacturer’s instructions. Values of sP-selectin concentrations were expressed in ng/mL; the lower detection limit was 15 ng/mL; the intra-assay CV was <10%; and the inter-assay CV was <15%. Values of GPVI were expressed as pg/mL; the lower detection limit was <11.72 pg/mL; the intra-assay CV was <8.0%; and the inter-assay CV was <10.0% [19].

Quantitative determination of serum inflammatory cytokine biomarkers interleukin-6 (IL-6) and tumour necrosis factor α (TNF-α) was performed with commercial ELISA immunoassays according to the manufacturer’s instructions (ELISA kit Thermo Fisher Scientific, Waltham, MA, USA). The limit of detection of human TNF-α was determined to be 1.65 pg/mL; the limit of detection of human IL-6 was determined to be 0.92 pg/mL. The CV was 6.2% intra-assay and 7.0% inter-assay.

Quantitative determination of serum levels of marinobufagenin was performed with commercial ELISA immunoassays according to the manufacturer’s instructions (ELISA kit MyBioSource, San Diego, CA, USA). The sensitivity of detection of human marinobufagenin was 0.1 nmol/L. The CV was <10.0% intra-assay and <12.0% inter-assay.

### 2.6. Proteomic Analysis

Proteomic analysis was performed on serum samples collected from a subset of 13 patients at baseline and after 6 months of follow-up. The selection criterion for the proteomic subanalysis was the sample quality. The samples were selected from those that showed no signs of hemolysis. The small sample size implies an exploratory, not confirmatory, analysis.

Five µL of serum were depleted of the fourteen most abundant proteins using the High-Select™ Top14 Abundant Protein™Depletion Mini Spin Columns (Thermo Fisher Scientific) following the manufacturer’s protocol. After depletion, the final volume of samples was 400 µL. Proteins in depleted samples were solubilized by adding 40 µL of Tris 1 M pH 8.5 and 16 µL of 10% SDS. Then, disulfide bonds between cysteines were reduced and alkylated by the addition of 45 µL of 100 mM dithiothreitol (DTT) and incubation for 5 min at 95 °C, followed by 54 µL of 200 mM iodoacetamide (IAA) and incubation for 1 h at 37 °C in the dark with gentle agitation; finally the excess of IAA was neutralised by a further addition of 9 µL of DTT 100 mM and incubation for 20 min at 37 °C with gentle agitation. Protein quantification was carried out on 10 µL of the obtained samples through Qubit fluorometric assay (Protein Kit, Thermo Fisher Scientific). Then, 5 mg were brought to a volume of 200 µL with a buffer composed of SDS 0.4%/Tris 100 mM, pH 8.5 and processed through the Protein Aggregation Capture protocol. Briefly, for each sample, 10 µL (200 µg) of MagResyn Hydroxyl beads (Resyn Biosciences, Pretoria, South Africa) was equilibrated by 2 sequential washes with 100 µL of 70% acetonitrile. Then, samples were added to the magnetic microparticles, and the precipitation of proteins was obtained by the addition of acetonitrile (ACN) to a final percentage of 70. The solution was incubated for 10 min at 1100 rpm. Three washes with 200 µL of CAN were followed by one wash with 70% ethanol. Finally, the beads were resuspended in 50 µL of triethylammonium bicarbonate and 50 mM Trypsin-Lys C protease mix (Thermo Fisher Scientific) was added at an E/S 1/25 (200 ng). After overnight incubation (37 °C, 1100 rpm), the peptide solution was harvested, and residual peptides were recovered by washing the beads with 50 µL of 0.1% formic acid. Forty µL of the digested peptides were purified by Strong Cation Exchange purification. Briefly, the resin was sequentially conditioned and equilibrated by wash A (20% ACN/0.5% formic acid (FA)) and wash B (80% ACN/0.5% FA). The peptide mixture was then loaded after being diluted to 200 µL in wash B. Thus, two washes (wash B, wash A) were performed. Peptides were eluted by adding 10 µL of the eluent solution (500 mM ammonium acetate/20% acetonitrile). Peptides were then lyophilized and resuspended in 40 µL of solution A (2% acetonitrile/0.1% formic acid); 4 µL were injected and analysed by nanoscale chromatography coupled to tandem mass spectrometry (nLC-MS/MS).

Mass spectrometric analysis was carried out on an Orbitrap Exploris 480 mass spectrometer (Thermo Fisher Scientific) operating in positive ion mode, coupled with an Easy LC 1200 nanoscale liquid chromatography system (Thermo Fisher Scientific). The analytical column was a pulled silica capillary of 15 cm (75 µm inner diameter) packed in-house with 3 µm C18 silica particles (Dr. Maisch). Nanoelectrospray (nESI) was obtained by applying a potential of 2000 V on the column front-end through a tee piece. Peptide elution was obtained through a binary gradient. In particular, mobile phase A contained 2% acetonitrile/0.1% formic acid (v:v) while mobile phase B was composed of 80% acetonitrile/ 0.1% formic acid. The flow rate was set to 300 nL/min. The total gradient duration was 140 min. In particular, the mobile phase B ramped from 3% to 25% in 90 min, from 25% to 40% in 30 min and from 40% to 100% in 8 min. Then, it was maintained for 10 min at 100%. The analytical column was equilibrated at 0% mobile phase B for 2 min. Data-independent acquisition (DIA) mode was used; the mass range of the full MS scan was from 370 to 900 *m*/*z* at a resolution of 60,000 (200 *m*/*z*). AGC target was 1 × 10^6^ and the maximum injection time was 50 ms. Twenty DIA scans were acquired at a resolution of 60,000 (200 *m*/*z*). The AGC target was 1 × 10^6^, the maximum injection time was 140 ms and the normalised collision energy was 25. Regarding the DIA scans, the isolation window was set to have 4 windows of 30 *m*/*z* (from 370 to 490), 12 windows of 20 *m*/*z* (from 490 to 740) and 3 windows of 50 *m*/*z* (from 750 to 900). The overlap was equal to 1 *m*/*z*. The resulting *m*/*z* range was 370–900. An aliquot from each sample was used to create a pool for spectral library building. The spectral library was realised through 10 runs having the same gradient and different scan acquisition and ranges. In more detail, 5 data-dependent acquisition (DDA1–5) runs were acquired with the following parameters: Full scan: Resolution 240,000 (200 *m*/*z*); maximum injection time 600 ms; AGC target 1 × 10^6^; number of dependent scans: 8; dynamic exclusion 20 s; MS/MS: Resolution 240,000 (200 *m*/*z*); maximum injection time 600 ms; AGC target 1 × 10^6^; isolation window 1.6 *m*/*z*; normalised collision energy 30%. The scan ranges were for DDA1–5: 370–470 *m*/*z*; 465–570 *m*/*z*; 565–670 *m*/*z*; 665–770 *m*/*z*; and 765–900 *m*/*z*. Five data-independent acquisition (DDA1–5) runs were acquired with the following parameters: The mass range for the full scan for DIA1 to DIA5 were, respectively, 370–470 *m*/*z*; 470–570 *m*/*z*; 570–670 *m*/*z*; 670–770 *m*/*z*; 770–900 *m*/*z*; Full scan settings: Resolution 60,000 (200 *m*/*z*); maximum injection time 50 ms; AGC target 1 × 10^6^; Twenty DIA scans of 5 *m*/*z* were acquired for DIA1–4, while 20 DIA scans of 6.5 *m*/*z* DIA were gained for DIA5. Normalised collision energy was set at 25. AGC target was 1 × 10^6^ and the maximum injection time was 120 ms. Raw files were searched by a hybrid search on Spectronaut software (version 18.7) against the database Human Reference Proteome (79,740 seq, downloaded on Uniprot in October 2022). Analysis settings were left as the default, modifying only the following parameters: precursor Qvalue and PEP cutoff and protein Qvalue and PEP were respectively set to 0.01. Only peptides shared between the proteins of the same protein group (protein group specific) were considered for quantification. Quantification was performed on the precursor identified in at least 70% of runs. Missing values were imputed using the background signal as an imputation strategy.

### 2.7. Statistical Analysis

Continuous variables were expressed as mean ± standard deviation (SD) (normally distributed data) or as median and interquartile range (IQR) (non-normally distributed data). Categorical data were expressed ass percentage. For all continuous variables, comparisons between baseline (T0) and post-treatment values (T6) were performed using a paired Student’s t-test for normally distributed data or Wilcoxon’s test for non-normally distributed data. All analyses were adjusted for sex. For categorical data, comparison was conducted by a chi-square test. Data for proteomic analysis were log transformed (log2) for protein intensity. The differences between baseline and follow-up were considered as Δlog2 and as fold changes (T0–T6). A linear correlation analysis was performed between variation in serum oxidative stress levels (ΔNox-2 and Δ8-isoprostane) and LVEF/GLS changes. Differences were assumed to be significant at *p* < 0.05. All comparisons were performed using SPSS 26.0 statistical software for Windows (SPSS, Inc, Chicago, IL, USA).

## 3. Results

After six months of therapeutic optimisation, we observed clinical and laboratory improvement, combined with selective changes in key proteins involved in inflammation, remodelling and endothelial function.

### 3.1. Baseline Population Characteristics

The study population included 100 HFimpEF patients (mean age 66.7 ± 11.8) with a predominantly male representation (n = 87). No significant differences were observed in the comparison between males and females for clinical parameters. The study cohort exhibited a substantial burden of CV comorbidities, with arterial hypertension being the most prevalent condition (60%), followed by ischemic heart disease (IHD) (65%) and atrial fibrillation (AF) (34%). Sleep apnea syndrome (SAS) was documented in 42% of the population. Metabolic comorbidities were frequent; in particular, type 2 diabetes mellitus (T2DM) was present in 51% of patients and dyslipidaemia in 48%. Chronic kidney disease (CKD) was observed in 26% of patients, while chronic obstructive pulmonary disease (COPD) affected 20% of patients in the study cohort.

Regarding pharmacological therapy, at baseline, 85 patients were in treatment with Beta-blockers, 83 with statins and 84 with antiplatelet agents. SGLT2 inhibitors were used in 43 patients, while 42 patients were in treatment with ARNI. Novel oral anticoagulants (NOACs) were administered to 27 patients, with only 3% receiving vitamin K antagonists. All patients completed the follow-up at six months after therapy improvement (Table 1).

### 3.2. Follow-Up Evaluation

After six months of follow-up, we observed significant improvements in glycometabolic profile, in particular, there was a significant reduction in fasting plasma glucose (FPG) levels (110.0 ± 34.8 mg/dL vs. 104.0 ± 25.0 mg/dL; *p* = 0.017), HbA1c (6.5 ± 0.9 vs. 6.3 ± 0.8; *p* = 0.019) and in fasting plasma insulin (FPI) (26.0 ± 11.3 μU/mL vs. 23.8 ± 8.8 μU/mL; *p* < 0.0001), suggesting improved insulin sensitivity. Moreover, regarding renal function parameters, there was a significant decrease in serum creatinine (1.1 ± 0.3 mg/dL vs. 1.0 ± 0.2 mg/dL; *p* = 0.022) and a significant increase in estimated glomerular filtration rate (eGFR) (74.0 ± 5.9 mL/min vs. 76.5 ± 8.6 mL/min; *p* = 0.046).

High-sensitivity C-reactive protein (Hs-CRP) showed a marked reduction (4.1 ± 1.4 mg/L vs. 3.0 ± 1.2 mg/L; *p* < 0.0001), indicating a significant decrease in systemic inflammation. NT-pro-BNP levels demonstrated a substantial decrease (2175.4 ± 648.4 pg/mL vs. 1785.7 ± 521.3 pg/mL; *p* < 0.0001), reflecting improved cardiac function and reduced cardiac stress (Table 2).

At the follow-up, there was a significant increase in LVEF (45.4 ± 9.3% vs. 48.9 ± 6.3%; *p* < 0.0001). GLS also showed significant improvement (−11.5 ± 1.6% vs. −12.4 ± 2.2%; *p* < 0.0001), indicating enhanced myocardial contractility. In addition, there was an improvement in diastolic function with a reduction in left ventricular filling pressures as evidenced by a reduction in E/e’ ratio (14.9 ± 4.7 vs. 14.3 ± 4.2; *p* = 0.028) and systemic congestion, as evidenced by inferior Vena Cava diameter (IVC) (19.2 ± 3.0 mm vs. 18.3 ± 2.3 mm; *p* < 0.0001). Right ventricular function showed improvement, with a significant increase in TAPSE (19.3 ± 4.0 mm vs. 19.8 ± 3.6 mm; *p* = 0.047). Moreover, there was a significant decrease in systolic pulmonary artery pressure (s-PAP) (39.6 ± 12.5 mmHg vs. 37.4 ± 10.3 mmHg; *p* = 0.048) (Table 3).

### 3.3. Inflammatory and Oxidative Stress Biomarkers

Significant reductions in pro-inflammatory cytokine levels were observed after six months of follow-up. We observed a significant decrease in IL-6 serum levels (2.7 ± 0.6 pg/mL vs. 2.4 ± 0.5 pg/mL; *p* < 0.0001, Δ = −11.11%) and TNF-α (29.0 ± 6.7 pg/mL vs. 24.7 ± 5.7 pg/mL; *p* < 0.0001, Δ = −14.8%).

Regarding oxidative stress biomarkers, there was a significant decrease in Nox-2 serum levels (0.7 ± 0.1 ng/mL vs. 0.5 ± 0.1 ng/mL; *p* < 0.0001, Δ = −28.6%) and 8-isoprostane levels (73.4 ± 10.8 pg/mL vs. 55.6 ± 10.1 pg/mL; *p* < 0.0001, Δ = −24.3%). Moreover, there was a significant reduction in MRBG (1.2 ± 0.2 vs. 1.0 ± 0.1; *p* < 0.0001, Δ = −16.7%).

In addition, platelet activation markers showed significant improvements; there was a significant decrease in sP-selectin serum levels (121.8 ± 19.7 ng/mL vs. 98.3 ± 15.7 ng/mL; *p* < 0.0001, Δ = −19.3%), indicating reduced platelet–endothelial interaction, and in GPVI serum levels (59.5 ± 12.0 ng/mL vs. 45.5 ± 11.1 ng/mL, *p* < 0.0001, Δ = −23.7%) (Table 4).

### 3.4. Proteomic Biomarkers Analysis

**Figure 1:**
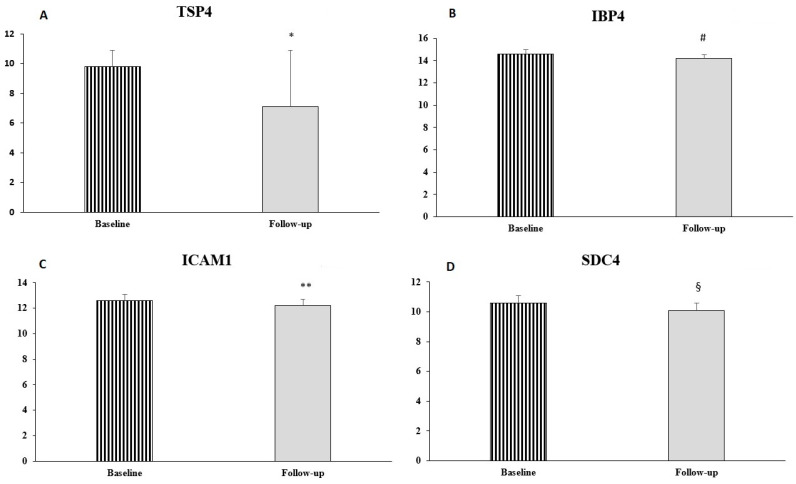
Graphical representation of protein levels at baseline and follow-up: TSP4 (**A**), IBP4 (**B**), ICAM1 (**C**), and SDC4 (**D**). Data are mean ± SD; * *p* = 0.028 vs. baseline, # *p* = 0.012 vs. baseline, ** *p* = 0.030 vs. baseline, § *p* = 0.005 vs. baseline. TSP4: thrombospondin-4; IBP4: insulin-like growth factor binding protein 4; ICAM1: intercellular adhesion molecule 1; SDC4: syndecan-4.

A comprehensive proteomic analysis, performed in a subgroup of 13 patients with HFimpEF, revealed selective changes in key CV-related proteins (Table 5). Insulin-like growth factor-binding protein 4 (IBP4) showed a significant decrease from 14.6 ± 0.4 to 14.2 ± 0.3 (*p* = 0.012, %var = −24.2%), suggesting alterations in growth factor signalling pathways. Thrombospondin-4 (TSP4), an extracellular matrix protein involved in vascular remodelling, decreased from 9.8 ± 1.1 to 7.1 ± 3.8 (*p* = 0.028, %var = −84.6%). Intercellular adhesion molecule 1 (ICAM1), a marker of endothelial activation, showed a modest but statistically significant increase from 12.6 ± 0.5 to 12.2 ± 0.5 (*p* = 0.030, %var = −24.2%). Syndecan-4 (SDC4), involved in cell adhesion and signalling, decreased significantly from 10.6 ± 0.5 to 10.1 ± 0.6 (*p* = 0.005, %var = −29.3%) (Table 6) (Figure 1).

Several other CV-related proteins showed numerical changes that did not reach statistical significance, including CD59 glycoprotein, interleukin-6 signal transducer (IL6RB), coagulation factor II (THRB), adiponectin (ADIPO), matrix metalloproteinases (MMP2, MMP9), and their inhibitor (TIMP1). While these changes were not statistically significant, they may represent biological trends worthy of further investigation in larger cohorts.

### 3.5. Correlation Analysis

A linear correlation analysis was performed to test the correlation between Δ values of oxidative stress biomarkers (ΔNox-2 and Δ8-isoprostane) and cardiac changes expressed as Δ variation between baseline and follow-up (ΔLVEF and ΔGLS). ΔNox-2 was correlated with ΔLVEF (r = −0.440, *p* = 0.005) and with ΔGLS (r = 0.410, *p* = 0.0014); Δ8-isoprostane was correlated with ΔLVEF (r = −0.380, *p* = 0.0032) and with ΔGLS (r = 0.350, *p* = 0.0071).

## 4. Discussion

The present study demonstrates significant improvements across multiple CV parameters in a cohort of 100 HFimpEF patients, providing compelling evidence for comprehensive therapeutic benefits targeting key pathophysiological mechanisms underlying HF progression. The observed improvements in cardiac function, neurohormonal activation, inflammatory cascades, oxidative stress pathways, and thrombotic risk represent a multi-dimensional therapeutic approach that addresses the complex interplay of mechanisms driving HF pathophysiology.

Obtained data demonstrated improvement in metabolic parameters, including HbA1c, fasting glucose levels and fasting insulin levels, indicating enhanced insulin sensitivity with potential CV benefits. Insulin resistance in HF contributes to impaired myocardial glucose utilisation, increased reliance on fatty acid oxidation with reduced cardiac efficiency, and enhanced oxidative stress generation through advanced glycation end-product formation [20].

The improvement in insulin sensitivity may enhance myocardial metabolic flexibility and allow more efficient substrate utilisation and improved cardiac energetics. This metabolic optimisation could contribute to the observed functional improvements through enhanced ATP synthesis and reduced oxidative stress burden. The substantial 28.6% reduction in NOX-2 levels observed in our study represents significant inhibition of vascular NADPH oxidase activity, a critical source of superoxide anion generation in both cardiomyocytes and vascular endothelial cells [21]. Nox-2-derived superoxide contributes to myocardial dysfunction through multiple mechanisms. First, ROS directly oxidises contractile proteins, particularly myosin heavy chains and troponin, leading to altered calcium sensitivity and impaired force generation [22]. This protein oxidation results in decreased contractile efficiency at the sarcomeric level, directly contributing to reduced EF. The reduction in Nox-2 activity suggests improved myocardial energetics and enhanced contractile function, which may contribute to the observed improvements in LVEF and GLS [18,21]. Additionally, reduced vascular Nox-2 activity would improve endothelial function through decreased oxidative inactivation of nitric oxide, potentially contributing to improved vasodilation and reduced afterload [23].

Moreover, the marked 24.3% reduction in 8-isoprostane indicates a substantial reduction in lipid peroxidation, particularly affecting arachidonic acid-containing membrane phospholipids [18]. 8-Isoprostanes are prostaglandin-like compounds formed through non-enzymatic, free radical-catalysed peroxidation of arachidonic acid in cellular membranes. 8-isoprostane not only serves as a reliable marker of oxidative stress but also exerts biological effects through thromboxane receptor activation, leading to vasoconstriction and enhanced platelet aggregation. Unlike many oxidative stress markers that require specific sample handling to prevent ex vivo artefact, 8-isoprostanes are chemically stable, making them reliable indicators of in vivo oxidative stress.

Mechanistically, 8-isoprostane exerts biological effects through activation of thromboxane A_2_ receptors (TP receptors) on vascular smooth muscle cells, platelets and endothelial cells.

The reduction in 8-isoprostane levels suggests improved membrane integrity in both cardiomyocytes and vascular cells, potentially contributing to enhanced cellular function and reduced thrombotic risk. This finding is particularly relevant in HF patients who demonstrate increased oxidative stress and elevated thrombotic risk [24].

In addition, significant reductions in sP-selectin and GPVI suggest decreased platelet activation and collagen-mediated aggregation, lowering thromboembolic risk. sP-selectin mediates the initial tethering and rolling of activated platelets on endothelium, representing the first step in thrombotic cascade activation [25]. The observed reduction suggests improved endothelial function and a decreased platelet activation state, which is particularly relevant in HF patients who demonstrate increased thrombotic risk through multiple mechanisms, including endothelial dysfunction, blood stasis, and hypercoagulability [26].

The reduction in GPVI levels suggests decreased platelet reactivity to vascular injury while potentially maintaining adequate haemostatic function. This finding may contribute to reduced microvascular thrombosis and improved tissue perfusion, particularly relevant given the increased atherothrombotic risk in heart failure patients [27].

Moreover, we observed a significant decrease in MRBG serum levels after six months of follow-up; high levels of MRBG have been shown to be associated with increased cardiac indices such as LVMI, left ventricular diastolic volume, and eccentric remodelling in patients with severe hypertension [28]. Furthermore, MRBG promotes vasoconstriction by inhibiting the Na^+^/K^+^-ATPase pump in vascular smooth muscle cells [29]. MRBG also activates numerous proliferative pathways, such as MAPK, which are also involved in determining cardiac fibrosis, such as phospholipase C [30].

Regarding inflammatory profile, the significant reductions in IL-6 and TNF-α represent coordinated suppression of key inflammatory mediators that drive HF progression through multiple pathophysiological mechanisms. IL-6 activates the JAK-STAT signalling pathway, leading to STAT3-mediated transcriptional changes that promote cardiomyocyte hypertrophy, interstitial fibrosis, and impaired contractile function [31]. The 11.1% reduction in IL-6 levels suggests decreased activation of this pathway, potentially contributing to the observed reverse remodelling and improved contractile performance. TNF-α exerts direct negative inotropic effects through sphingomyelinase activation and ceramide production, resulting in impaired calcium handling and contractile dysfunction [32]. Additionally, TNF-α promotes cardiomyocyte apoptosis through mitochondrial dysfunction and caspase activation, contributing to progressive myocardial loss. The 14.8% reduction in TNF-α levels may directly contribute to improved contractility and reduced cardiomyocyte death.

The anti-inflammatory profile observed in this study is particularly relevant given the established role of inflammation in HF progression [33]. Chronic inflammatory activation contributes to endothelial dysfunction, accelerated atherosclerosis, insulin resistance, and direct myocardial toxicity. The multi-cytokine suppression observed suggests a comprehensive anti-inflammatory effect that may contribute to the observed improvements in cardiac function and metabolic parameters.

Of particular interest, the proteomic analysis carried out in the present study in a subgroup of 13 elderly patients with HFimpEF provided insights into specific mechanistic pathways involved in disease progression and therapeutic response.

The significant reduction in IBP4 may reflect altered IGF-1 bioavailability and signalling [34]. IBP4 is one of the major binding proteins that sequesters IGF-1, thereby limiting its interaction with the IGF-1 receptor. By inhibiting IGF-1 activity, IBP4 reduces downstream cardioprotective effects such as enhanced glucose uptake, protein synthesis, mitochondrial function, and anti-apoptotic signalling. IBP4 primarily inhibits IGF-1 activity, and its reduction could increase IGF-1 bioavailability, potentially enhancing cardiac metabolism, protein synthesis, and anti-apoptotic signalling. Elevated IBP4 levels have been associated with adverse CV outcomes, as excessive sequestration of IGF-1 can impair myocardial repair and adaptation. Therefore, the observed decrease in IBP4 may increase IGF-1 bioavailability, supporting cardiomyocyte survival, contractile performance, and improved myocardial energetics [34,35].

Similarly, we observed a significant decrease in TSP4 after six months of follow-up; TSP4 is a matricellular glycoprotein expressed at low levels in the myocardium under physiological conditions. Its expression is markedly upregulated in response to biomechanical stress, pressure overload, ischemia, and inflammatory stimuli [36]. Mechanistically, TSP4 participates in extracellular matrix (ECM) remodelling, cell–matrix communication, and endoplasmic reticulum (ER) stress modulation. In the early stages of cardiac stress, TSP4 exerts protective effects by stabilising ECM architecture and attenuating ER stress responses, thus supporting cardiomyocyte survival [37]. However, chronic and sustained overexpression of TSP4 promotes maladaptive ECM deposition, fibrosis, and myocardial stiffening, leading to impaired diastolic relaxation and adverse remodelling [38]. The reduction in TSP4 levels observed after therapeutic optimisation in our study may therefore reflect attenuation of maladaptive fibrotic signalling and improved balance between protective and pathological ECM remodelling processes. These findings underscore the pathophysiological relevance of both IBP4 and TSP4 in the context of heart failure. By modulating growth factor signalling and ECM remodelling, respectively, they represent promising biomarkers for disease progression and potential therapeutic targets aimed at restoring homeostatic cardiac adaptation while preventing maladaptive remodelling.

In addition, data obtained from proteomic analysis also highlighted a decrease in ICAM-1, a key adhesion molecule involved in endothelial activation and inflammatory responses [39].

ICAM-1 is normally expressed at low levels on endothelial cells, but is markedly upregulated by pro-inflammatory cytokines such as TNF-α, IL-1β, and IFN-γ [40]. Mechanistically, ICAM-1 mediates leukocyte adhesion and transmigration through interaction with β2 integrins (LFA-1, Mac-1), thereby facilitating immune cell infiltration into the myocardium [41]. This process contributes to chronic myocardial inflammation, microvascular dysfunction, and subsequent adverse remodelling. Moreover, persistent ICAM-1 expression promotes fibroblast activation and extracellular matrix deposition, leading to progressive myocardial fibrosis and stiffening, with implications for both systolic and diastolic dysfunction [42]. Thus, the observed reduction in ICAM-1 expression after therapy optimisation in our cohort may reflect an attenuation of endothelial activation and inflammatory signalling, ultimately contributing to improved ventricular remodelling and clinical stability.

Moreover, the significant reduction in SDC4 represents a mechanistically important change in vascular homeostasis and inflammatory regulation. Syndecan-4 is a transmembrane heparan sulphate proteoglycan that functions as a co-receptor for multiple signalling pathways involved in CV pathophysiology. At the molecular level, SDC4 interacts with integrin receptors to modulate focal adhesion formation and cytoskeletal reorganisation, processes critical for endothelial barrier integrity and vascular permeability [43]. Under pathological conditions, elevated SDC4 expression promotes endothelial cell activation through enhanced NF-κB signalling, leading to increased expression of adhesion molecules (VCAM-1, ICAM-1) and pro-inflammatory cytokines (IL-6, TNF-α). Elevated shear stress and inflammatory stimuli upregulate SDC4 expression, which leads to enhanced production of reactive oxygen species via NADPH oxidase activation and increased expression of matrix metalloproteinases (MMP-2, MMP-9), contributing to vascular remodelling and atherosclerotic plaque instability [44]. Furthermore, SDC4 plays a critical role in platelet–endothelial interactions by serving as a docking platform for P-selectin and facilitating platelet adhesion to activated endothelium. Elevated SDC4 levels enhance the binding affinity of platelets to endothelial cells, promoting thrombotic events and microvascular dysfunction characteristic of HF.

The observed reduction in SDC4 levels suggests multiple beneficial effects: decreased endothelial permeability through stabilised intercellular junctions, reduced inflammatory signalling via dampened NF-κB activation, improved vascular compliance through decreased matrix metalloproteinase activity, and reduced thrombotic potential through diminished platelet-endothelial adhesion. These mechanisms collectively contribute to improved endothelial function, reduced vascular inflammation, and enhanced microvascular perfusion, mechanistically supporting the observed improvements in cardiac hemodynamic and systemic inflammatory markers.

Several limitations should be acknowledged in interpreting these results. The main limitations are the lack of a control group and the very small size of the proteomic analysis.

Another limitation of the study is that enrolled subjects are geriatric outpatients. The relatively short follow-up period may not capture long-term effects or potential adverse consequences of the observed changes. Moreover, the absence of classical antioxidant defence markers (superoxide dismutase, glutathione peroxidase, and catalase) represents another limitation of the current study.

The proteomic analysis, while providing mechanistic insights, requires validation in larger cohorts and correlation with functional outcomes. Some protein changes, such as the increase in ICAM-1, warrant careful monitoring to ensure they do not represent early markers of adverse effects.

## 5. Conclusions

This study demonstrates significant improvements across multiple CV biomarkers, after six months of therapy optimisation, in HF patients, providing evidence for comprehensive therapeutic effects targeting inflammation, oxidative stress, neurohormonal activation, and thrombotic risk. The multi-pathway therapeutic effects address key pathophysiological mechanisms underlying HF progression, suggesting a disease-modifying rather than merely symptomatic approach.

The mechanistic insights provided by the multi-biomarker approach support the biological plausibility of the observed functional improvements and suggest potential disease-modifying effects in HF pathophysiology. The magnitude and consistency of improvements across diverse pathways indicate a holistic therapeutic approach that addresses the complex, interconnected mechanisms driving HF progression.

Beyond the cross-sectional evaluation of oxidative stress biomarkers at a single follow-up timepoint, there is growing interest in the concept of dynamic biomarker monitoring as a strategy to guide therapeutic decisions and risk stratification in HFimpEF. Taken together, the integration of Nox-2 and 8-isoprostane into a longitudinal, multi-timepoint monitoring strategy—extending beyond the six-month window evaluated in the present study—could represent a clinically meaningful approach to identify HFimpEF patients at risk of incomplete biological recovery, to individualise and titrate antioxidant and cardioprotective therapies, and to define thresholds for escalation of treatment. Future prospective studies with extended follow-ups are warranted to validate these biomarkers as dynamic prognostic tools in the management of HFimpEF.

Future research should focus on correlating these biomarker changes with patient-reported outcomes, functional capacity measures, and long-term clinical events. Additionally, investigation of the temporal relationships between different biomarker changes may provide insights into the sequence of therapeutic effects and optimal monitoring strategies.

## Figures and Tables

**Table 1:** Comorbidities and pharmacotherapy (baseline and follow-up) of study population.

	Whole Population (n = 100)	
**Male gender**, n (%)	87 (87%)	
**Age (yrs)**	66.7	
**Arterial hypertension**, n (%)	60 (60%)	
**AF**, n (%)	34 (34%)	
**Dislipidemia**, n (%)	48 (48%)	
**SAS**, n (%)	42 (42%)	
**IHD**, n (%)	65 (65%)	
**Obesity**, n (%)	23 (23%)	
**VHD**, n (%)	42 (42%)	
**CKD**, n (%)	26 (26%)	
**COPD**, n (%)	20 (20%)	
**T2DM**, n (%)	51 (51%)	
**Smokers**, n (%)	20 (20%)	
***NYHA class***		
**NYHA class II**	70	
**NYHA class III**	30	
	**Baseline**	**FU**
**β-blockers**, n	85	84
**ACEi/ARBs**, n	44	38
**SGLT2i**, n	43	80
**MRAs**, n	30	80
**ARNI**, n	42	50
**GLP-1RA**, n	13	16
**Insulin**, n	10	11
**Statins**, n	83	85
**Diuretics**, n	65	70
**Antiplatelets drug**, n	84	84
**VKAs**, n	12	3
**NOACs**, n	27	34

AF: atrial fibrillation; SAS: sleep apnoea syndrome; IHD: ischemic heart disease; VHD: Valvular Heart Disease; CKD: chronic kidney disease; COPD: chronic obstructive pulmonary disease; T2DM: Diabetes Mellitus Type 2; ACEi: angiotensin-converting enzyme inhibitors; ARB: angiotensin II receptor blockers; ARNI: angiotensin receptor neprilysin inhibitor; MRAs: mineralocorticoid receptor antagonists; SGLT2i: sodium-glucose cotransporter 2 inhibitors; GLP-1 RA: glucagon-like-peptide 1 receptor agonists; VKAs: vitamin K antagonists; NOACs: non-vitamin K antagonist oral anticoagulants.

**Table 2:** Comparison of baseline and follow-up on clinical, haemodynamic and laboratory parameters.

	Baseline	Follow Up	*p*
**BMI**, kg/m^2^	30.0 ± 5.6	30.1 ± 4.8	0.823
**SBP**, mmHg	124.5 ± 13.9	123.9 ± 14.5	0.595
**DBP**, mmHg	74.1 ± 9.0	74.4 ± 8.5	0.695
**Na**, mmol/L	141.0 ± 2.6	141.0 ± 2.8	0.934
**K**, mmol/L	4.5 ± 0.4	4.5 ± 0.4	0.801
**FPG**, mg/dL	110.0 ± 34.8	104.0 ± 25.0	0.017
**FPI**, μU/mL	26.0 ± 11.3	23.8 ± 8.8	<0.0001
**HbA1c**, (%)	6.5 ± 0.9	6.3 ± 0.8	0.019
**Albumin**, mg/dL	4.0 ± 0.6	4.0 ± 0.7	0.817
**Vitamin D**, ng/mL	27.5 ± 8.9	29.7 ± 8.6	0.027
**Creatinine**, mg/dL	1.1 ± 0.3	1.0 ± 0.2	0.022
***eGFR***, mL/min	74.0 ± 5.9	76.5 ± 8.6	0.046
**PLT**, 10^3^/mm^3^	208.8 ± 48.6	201.4 ± 45.2	0.052
**LDL**, mg/dL	66.0 ± 29.2	63.8 ± 24.0	0.443
**HDL**, mg/dL	45.8 ± 10.3	46.0 ± 9.5	0.948
**Triglycerides**, mg/dL	113.6 ± 39.8	115.6 ± 36.5	0.533
**hs-CRP** (mg/L)	4.1 ± 1.4	3.0 ± 1.2	<0.0001
**Uric acid** (mg/dL)	7.5 ± 0.7	5.3 ± 1.3	0.427
**NT-pro-BNP** (pg/mL)	2175.4 ± 648.4	1785.7 ± 521.3	<0.0001

BMI: body mass index; SBP: systolic blood pressure, DBP: diastolic blood pressure; FPG: fasting plasma glucose; FPI: fasting plasma insulin; eGFR: estimate glomerular filtration rate; PLT: platelet count; Na: sodium; K: potassium; LDL: low density lipoproteins; HDL: high-density lipoprotein; hs-CRP: high-sensitivity C-reactive protein; NT-pro-BNP: N-terminal pro-brain natriuretic peptide.

**Table 3:** Echocardiographic parameters of the study population at baseline and follow-up in the whole study population.

	Baseline	Follow Up	*p*
**LAVI**, mL/m^2^	44.1 ± 11.5	44.0 ± 12.6	0.933
**LVESV/BSA**, mL/m^2^	82.2 ± 16.0	79.8 ± 14.3	0.058
**LVEF**, %	45.4 ± 9.3	48.9 ± 6.3	<0.0001
**CI**, mL/min/1.73 m^2^	1883.7 ± 192.2	1906.3 ± 237.6	0.181
**E/A**	1.1 ± 0.5	1.1 ± 0.4	0.232
**E/e’**	14.9 ± 4.7	14.3 ± 4.2	0.028
**GLS**, %	−11.5 ± 1.6	−12.4 ± 2.2	<0.0001
**RVOTp**, m/s	2.8 ± 0.5	2.7 ± 0.5	0.195
**TAPSE**, mm	19.3 ± 4.0	19.8 ± 3.6	0.047
**s-PAP**, mmHg	39.6 ± 12.5	37.4 ± 10.3	0.048
**IVC**, mm	19.2 ± 3.0	18.3 ± 2.3	<0.0001
**TAPSE/s-PAP**, mm/mmHg	0.6 ± 0.2	0.6 ± 0.2	0.310

LAVI: left atrial volume index; LVESV/BSA: left ventricular end-systolic volume indexed to body surface area; LVEF: left ventricular ejection fraction; CI: cardiac index; E/A: ratio between wave E (the wave of rapid filling in early diastole) and wave A (the wave of atrial contraction); E/e’: between wave E and wave e′ (reliable estimate of changes in end-diastolic blood pressure); GLS: global longitudinal strain; RVOTp: right ventricular outflow tract proximal; TAPSE: tricuspid annular plane systolic excursion; s-PAP: systolic pulmonary arterial pressure; IVC: inferior vena cava.

**Table 4:** Changes in oxidative stress, platelet activation and inflammatory cytokines between baseline and follow-up, in the whole study population.

	Baseline	Follow Up	*p*	Δ(T0T6)
**IL-6**	2.7 ± 0.6	2.4 ± 0.5	<0.0001	−11.1%
**TNF-α**	29.0 ± 6.7	24.7 ± 5.7	<0.0001	−14.8%
**MRBG**	1.2 ± 0.2	1.0 ± 0.1	<0.0001	−16.7%
**Nox-2**	0.7 ± 0.1	0.5 ± 0.1	<0.0001	−28.6%
**8-isoprostane**	73.4 ± 10.8	55.6 ± 10.1	<0.0001	−24.2%
**Sp-selectin**	121.8 ± 19.7	98.3 ± 15.7	<0.0001	−19.3%
**GPVI**	59.5 ± 12.0	45.5 ± 11.1	<0.0001	−23.5%

IL-6: Interleukin-6; TNF-α: tumour necrosis factor alpha; MRBG: marinobufagenin; Nox-2: NADPH oxidase 2; GPVI: Glycoprotein VI.

**Table 5:** List of the proteins suggested as therapeutic target biomarkers in heart failure.

Protein Name	UniProt ID	Gene	Description
CAH2	P00918	*CA2*	Carbonic anhydrase 2
IBP4	P22692	*IGFBP4*	Insulin-like growth factor binding protein 4
B1AHL2	B1AHL2	*FBLN1*	Fibulin-1
CD59 glycoprotein	P13987	*CD59*	CD59 glycoprotein
TSP4	P35443	*THBS4*	Thrombospondin-4
CNTN1	Q12860	*CNTN1*	Contactin-1
LG3BP	Q08380	*LGALS3BP*	Galectin-3-binding protein
IL6RB	P40189	*IL6ST*	Interleukin-6 receptor subunit beta
THRB	P00734	*F2*	Prothrombin
PERM	P05164	*MPO*	Myeloperoxidase
ADIPO	Q15848	*Adipoq*	Adiponectin
MMP2	P08253	*MMP2*	Matrix metalloproteinase-2
MMP9	P14780	*MMP9*	Matrix metalloproteinase-9
TIMP1	P01033	*TIMP*	Metalloproteinase inhibitor 1
IGF1	P05019	*IGF1*	Insulin-like growth factor I
ICAM1	P05362	*ICAM1*	Intercellular adhesion molecule 1
VCAM1	P19320	*VCAM1*	Vascular cell adhesion protein 1
SDC4	P31431	*SDC4*	Syndecan-4
CYTC	P01034	*CST3*	Cystatin-C

**Table 6:** Proteomic analysis in a subgroup of 13 patients with HFimpEF.

Protein Name	Baseline	Follow Up	*p*	Δlog2 (T0-T6)	%Variation
CAH2	15.3 ± 1.2	14.4 ± 1.8	0.226	−0.9	−46.4%
IBP4	14.6 ± 0.4	14.2 ± 0.3	0.012	−0.4	−24.2%
B1AHL2	14.7 ± 0.7	14.4 ± 0.6	0.338	−0.3	−18.8%
CD59 glycoprotein	14.4 ± 0.9	13.7 ± 0.9	0.067	−0.7	−38.4%
TSP4	9.8 ± 1.1	7.1 ± 3.8	0.028	−2.7	−84.6%
CNTN1	13.5 ± 0.6	13.4 ± 0.5	0.564	−0.1	−6.7%
LG3BP	18.6 ± 0.5	18.5 ± 0.5	0.814	−0.1	−6.7%
IL6RB	12.6 ± 0.4	12.4 ± 0.3	0.057	−0.2	−12.9%
THRB	18.1 ± 0.9	17.6 ± 0.6	0.124	−0.5	−29.3%
PERM	12.7 ± 0.9	12.4 ± 1.0	0.517	−0.3	−18.8%
ADIPO	16.9 ± 0.7	17.4 ± 0.9	0.137	+0.5	+41.4%
MMP2	13.4 ± 0.6	13.2 ± 0.4	0.304	−0.2	−12.9%
MMP9	14.1 ± 0.6	13.8 ± 1.0	0.456	−0.3	−18.8%
TIMP1	13.6 ± 0.5	13.4 ± 0.7	0.211	−0.2	−12.9%
IGF1	13.6 ± 0.7	14.0 ± 0.7	0.107	+0.4	+31.9%
ICAM1	12.6 ± 0.5	12.2 ± 0.5	0.030	−0.4	−24.2%
VCAM1	14.4 ± 0.5	14.3 ± 0.4	0.689	−0.1	−6.7%
SDC4	10.6 ± 0.5	10.1 ± 0.6	0.005	−0.5	−29.3%
CYTC	16.9 ± 0.6	16.6 ± 0.4	0.070	−0.3	−18.8%

Values of protein intensity are log transformed (log2) and expressed as mean ± DS. CAH2: carbonic anhydrase 2; IBP4: insulin-like growth factor binding protein 4; B1AHL2: Fibulin-1; TSP4: Thrombospondin-4; CNTN1: Contactin-1; LG3BP: Galectin-3-binding protein; IL6RB: Interleukin-6 receptor subunit beta; THRB: Prothrombin; PERM: Myeloperoxidase; ADIPO: Adiponectin; MMP2: matrix metalloproteinase-2; MMP9: matrix metalloproteinase-9; TIMP1: metalloproteinase inhibitor 1; IGF1: insulin-like growth factor I; ICAM1: intercellular adhesion molecule 1; VCAM1: vascular cell adhesion protein 1; SDC4: Syndecan-4; CYTC: Cystatin-C.

## Data Availability

The original contributions presented in this study are included in the article. Further inquiries can be directed to the corresponding authors.

## Abbreviations

**Abbreviation**	**Definition**
e-GFR	Estimated glomerular filtration rate
DTT	dithiothreitol
IAA	iodoacetamide
ACT	acetonitrile
DIA	Data-independent acquisition
DDA1–5	5 data-dependent acquisition
SD	standard deviation
IQR	interquartile range
T2DM	type 2 diabetes mellitus
ICH	ischemic heart disease
SAS	Sleep apnoea syndrome
AF	atrial fibrillation
CKD	Chronic kidney disease
COPD	chronic obstructive pulmonary disease
NOACs	Novel oral anticoagulants
FPG	fasting plasma glucose
IL6RB	interleukin-6 signal transducer
THRB	coagulation factor II
ADIPO	adiponectin
MMP2	matrix metalloproteinase 2
MMP9	matrix metalloproteinases 9
ECM	extracellular matrix
ER	Endoplasmic reticulum
HF	Heart Failure
NT-proBNP	N-terminal pro-B-type natriuretic peptide
HFimEF	Heart Failure with improved ejection fraction
LVEF	Left Ventricular Ejection Fraction
CV	Cardiovascular
IBP4	Insulin-like growth factor-binding protein 4
TSP4	Thrombospondin-4
ICAM1	Intercellular adhesion molecule 1
SDC4	Syndecan-4
HFrEF	Heart failure with reduced ejection fraction
GCP	Good clinical practice
MAGIC-HF	MAgna GraecIa evaluation of Comorbidities in patients with Heart Failure
BP	Blood Pressure
PP	Pulse pressure
LVM	Left ventricular mass
BSA	body surface area
CI	Cardiac Index
RVOT	Right ventricular outflow tract
RAA	Right atrium area
TAPSE	Tricuspid annular plane systolic excursion
